# Childhood-Onset Schizophrenia Spectrum Disorders: Neurodevelopmental Influences on Psychotic Symptoms and Psychopathological Profiles

**DOI:** 10.1192/j.eurpsy.2026.10577

**Published:** 2026-07-31

**Authors:** M. Di Luzio, M. Pontillo, I. Bertoncini, B. D’Aiello, R. Averna, M. Labonia, S. Vicari

**Affiliations:** 1 Child and Adolescent Neuropsychiatry Unit, Bambino Gesù Children’s Hospital, IRCCS; 2 Life Sciences and Public Health Department, Catholic University, Rome, Italy

## Abstract

**Introduction:**

Childhood-Onset Schizophrenia Spectrum Disorder (COSS) represent a very early-onset subtype of schizophrenia spectrum disorders, typically manifesting before age 13. Although interest in COSS has grown in recent years, studies exploring its clinical characteristics remain scarce.

**Objectives:**

This study aimed to analyze clinical differences within the COSS group based on the presence of psychiatric or neurodevelopmental comorbidities to delineate distinct psychopathological profiles of childhood psychosis. We hypothesized that COSS presents variable symptomatology depending on the presence of comorbid psychiatric disorders, neurodevelopmental disorders, or the absence of comorbidities.

**Methods:**

Seventy children diagnosed with COSS were selected at the onset of psychotic symptoms from admission to a specialized very early-onset psychosis clinical service. Participants were assessed and divided into three subgroups based on comorbidity profiles: COSS with neurodevelopment disorder comorbidity, COSS with psychiatric disorder comorbidity, COSS without psychiatric or neurodevelopmental disorder comorbidities. Psychotic symptoms were indexed using the Structured Interview for Psychosis-Risk Syndrome.

**Results:**

The Statistical analysis of the subgroups revealed that COSS with neurodevelopmental disorder comorbidity exhibited a distinct profile, characterized by earlier onset of psychotic symptoms, lower intelligence quotient, greater disorganization symptoms, and fewer negative symptoms compared to COSS with psychiatric disorder comorbidity. COSS with psychiatric disorder comorbidity and COSS without comorbidities showed more similarities with each other than with COSS with neurodevelopmental disorder comorbidity, suggesting a significant influence of neurodevelopmental comorbidities on clinical presentation.

**Image 1:**
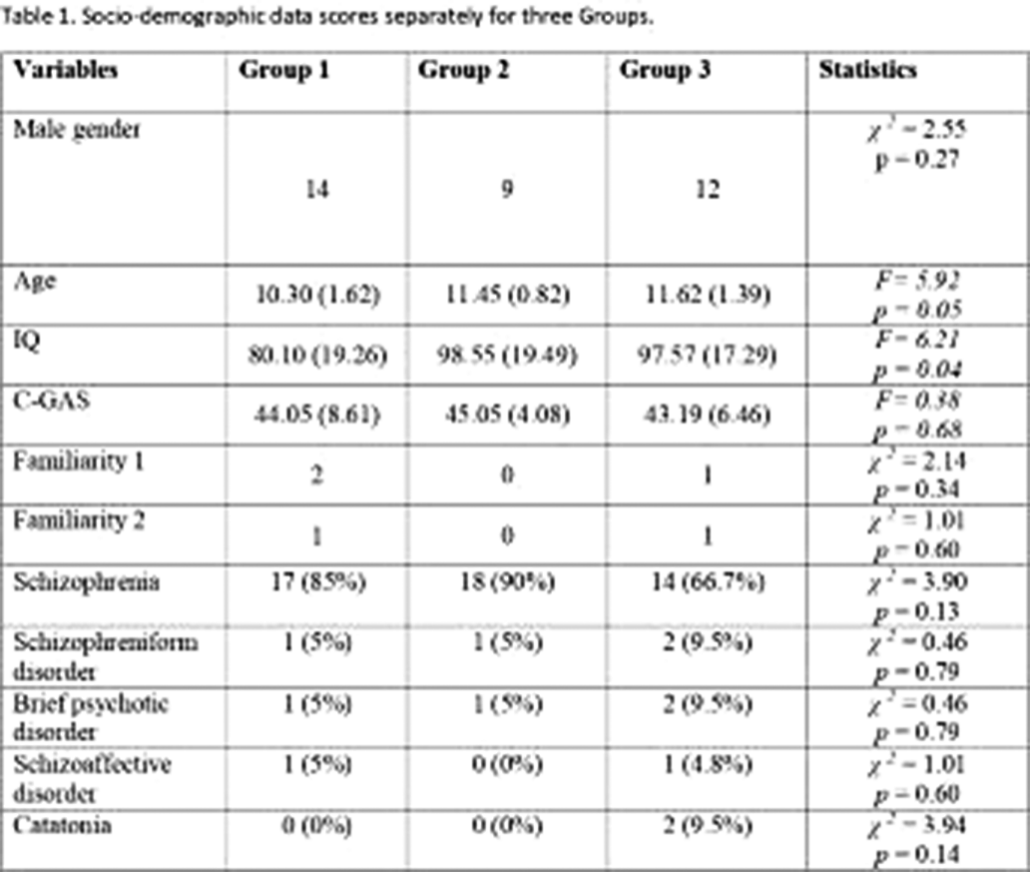
Image 1: Long description.

**Image 2:**
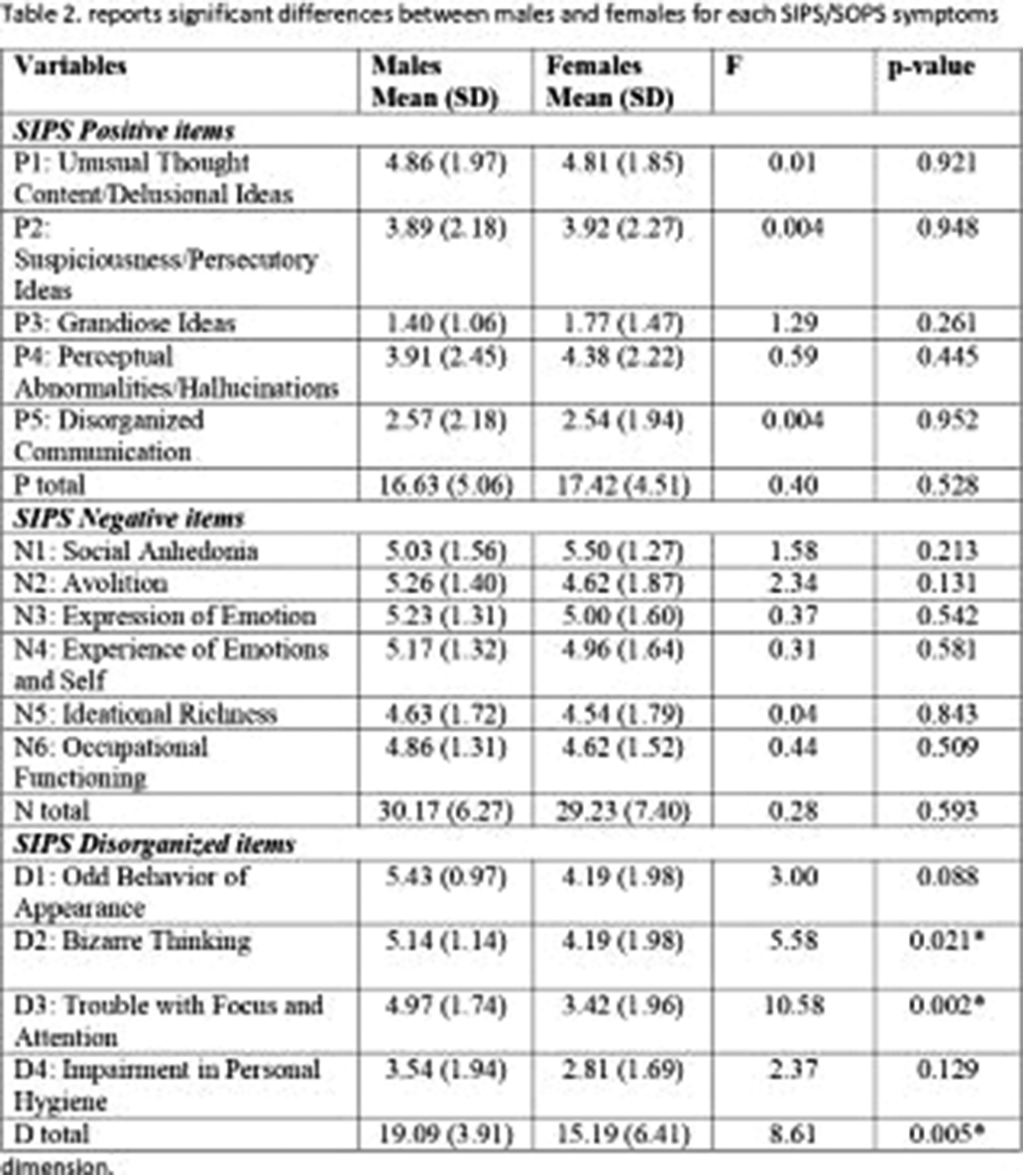
Image 2: Long description.

**Image 3:**
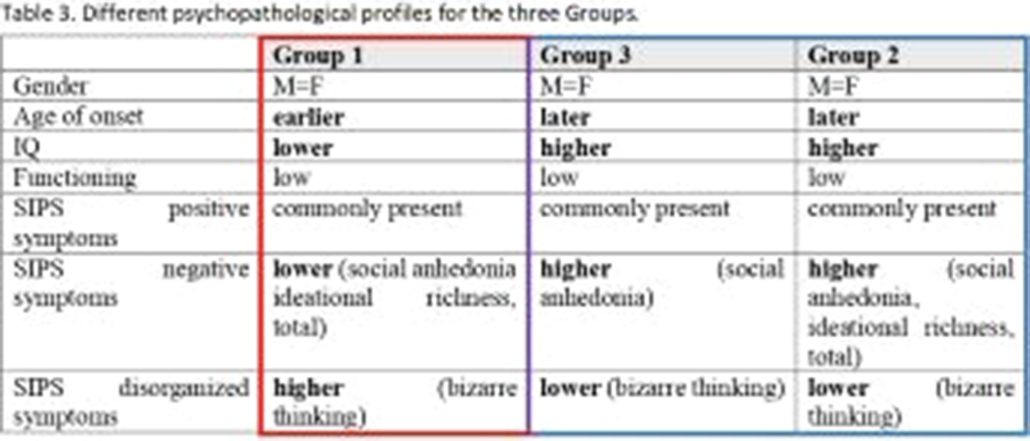
Image 3: Long description.

**Conclusions:**

Identifying specific psychopatological profiles within COSS may enhance personalized treatment approaches for COSS, as well as for early-onset and adult-onset schizophrenia spectrum disorders.

**Disclosure of Interest:**

None Declared

## Long descriptions

### Image 1: Long description

The table consists of five columns: Variables, Group 1, Group 2, Group 3, and Statistics.

* Male gender: Group 1 has 14, Group 2 has 9, Group 3 has 12. Statistics: chi-squared = 2.55, p = 0.27.

* Age: Group 1 is 10.30 (1.62), Group 2 is 11.45 (0.82), Group 3 is 11.62 (1.39). Statistics: F = 5.92, p = 0.05.

* I Q: Group 1 is 80.10 (19.26), Group 2 is 98.55 (19.49), Group 3 is 97.57 (17.29). Statistics: F = 6.21, p = 0.04.

* C-GAS: Group 1 is 44.05 (8.61), Group 2 is 45.05 (4.08), Group 3 is 43.19 (6.46). Statistics: F = 0.38, p = 0.68.

* Familiarity 1: Group 1 is 2, Group 2 is 0, Group 3 is 1. Statistics: chi-squared = 2.14, p = 0.34.

* Familiarity 2: Group 1 is 1, Group 2 is 0, Group 3 is 1. Statistics: chi-squared = 1.01, p = 0.60.

* Schizophrenia: Group 1 is 17 (85%), Group 2 is 18 (90%), Group 3 is 14 (66.7%). Statistics: chi-squared = 3.90, p = 0.13.

* Schizophreniform disorder: Group 1 is 1 (5%), Group 2 is 1 (5%), Group 3 is 2 (9.5%). Statistics: chi-squared = 0.46, p = 0.79.

* Brief psychotic disorder: Group 1 is 1 (5%), Group 2 is 1 (5%), Group 3 is 2 (9.5%). Statistics: chi-squared = 0.46, p = 0.79.

* Schizoaffective disorder: Group 1 is 1 (5%), Group 2 is 0 (0%), Group 3 is 1 (4.8%). Statistics: chi-squared = 1.01, p = 0.60.

* Catatonia: Group 1 is 0 (0%), Group 2 is 0 (0%), Group 3 is 2 (9.5%). Statistics: chi-squared = 3.94, p = 0.14.

Navigate back to Image 1:.

### Image 2: Long description

The table is titled Table 2 and contains five columns: Variables, Males Mean (S D), Females Mean (S D), F, and p-value. It is divided into three thematic sections.

1. S I P S Positive items:

- P 1 Unusual Thought Content/Delusional Ideas: Males 4.86 (1.97), Females 4.81 (1.85), F 0.01, p 0.921.

- P 2 Suspiciousness/Persecutory Ideas: Males 3.89 (2.18), Females 3.92 (2.27), F 0.004, p 0.948.

- P 3 Grandiose Ideas: Males 1.40 (1.06), Females 1.77 (1.47), F 1.29, p 0.261.

- P 4 Perceptual Abnormalities/Hallucinations: Males 3.91 (2.45), Females 4.38 (2.22), F 0.59, p 0.445.

- P 5 Disorganized Communication: Males 2.57 (2.18), Females 2.54 (1.94), F 0.004, p 0.952.

- P total: Males 16.63 (5.06), Females 17.42 (4.51), F 0.40, p 0.528.

2. S I P S Negative items:

- N 1 Social Anhedonia: Males 5.03 (1.56), Females 5.50 (1.27), F 1.58, p 0.213.

- N 2 Avolition: Males 5.26 (1.40), Females 4.62 (1.87), F 2.34, p 0.131.

- N 3 Expression of Emotion: Males 5.23 (1.31), Females 5.00 (1.60), F 0.37, p 0.542.

- N 4 Experience of Emotions and Self: Males 5.17 (1.32), Females 4.96 (1.64), F 0.31, p 0.581.

- N 5 Ideational Richness: Males 4.63 (1.72), Females 4.54 (1.79), F 0.04, p 0.843.

- N 6 Occupational Functioning: Males 4.86 (1.31), Females 4.62 (1.52), F 0.44, p 0.509.

- N total: Males 30.17 (6.27), Females 29.23 (7.40), F 0.28, p 0.593.

3. S I P S Disorganized items:

- D 1 Odd Behavior of Appearance: Males 5.43 (0.97), Females 4.19 (1.98), F 3.00, p 0.088.

- D 2 Bizarre Thinking: Males 5.14 (1.14), Females 4.19 (1.98), F 5.58, p 0.021 with asterisk.

- D 3 Trouble with Focus and Attention: Males 4.97 (1.74), Females 3.42 (1.96), F 10.58, p 0.002 with asterisk.

- D 4 Impairment in Personal Hygiene: Males 3.54 (1.94), Females 2.81 (1.69), F 2.37, p 0.129.

- D total: Males 19.09 (3.91), Females 15.19 (6.41), F 8.61, p 0.005 with asterisk.

Navigate back to Image 2:.

### Image 3: Long description

The table compares Group 1 (outlined in red), Group 3 (outlined in purple), and Group 2 (outlined in blue) across seven rows of data.

* Gender: All three groups show M equals F.

* Age of onset: Group 1 is earlier, while Group 3 and Group 2 are later.

* I Q: Group 1 is lower, while Group 3 and Group 2 are higher.

* Functioning: All three groups are categorized as low.

* S I P S positive symptoms: All three groups are categorized as commonly present.

* S I P S negative symptoms: Group 1 is lower (social anhedonia, ideational richness, total). Group 3 is higher (social anhedonia). Group 2 is higher (social anhedonia, ideational richness, total).

* S I P S disorganized symptoms: Group 1 is higher (bizarre thinking). Group 3 is lower (bizarre thinking). Group 2 is lower (bizarre thinking).

Navigate back to Image 3:.

